# Physical Activity, Sleep, and Demographic Patterns in Alaska Native Children and Youth Living in Anaktuvuk Pass

**DOI:** 10.3390/youth3010021

**Published:** 2023-02-24

**Authors:** Vernon Grant, Deborah Mekiana, Jacques Philip

**Affiliations:** 1 Center for American Indian and Rural Health Equity, Montana State University, Bozeman, MT 59717, USA; 2 Alaska Native Studies, University of Alaska, Fairbanks, AK 99775, USA; 3 Center for Alaska Native Health Research, University of Alaska Fairbanks, Fairbanks, AK 99775, USA

**Keywords:** youth, physical activity, sleep, Alaska Native community

## Abstract

Physical activity (PA), sleep, and weight are important factors for youth health. However, data about these factors are unknown in youth living in isolated Alaska Native communities. This study aims to assess PA, sleep, height and weight in elementary through high school students living in Anaktuvuk Pass. Fourteen children (<12) and 24 youths (12–20) volunteered to participate in this study. PA and sleep data were collected with actigraphy. Height and weight were assessed with standard procedures. Demographics were collected via survey. Results show that 10.53% and 18.42% of participants were overweight and obese, respectively. Average bedtime was 00:15 am and wake time 08:23 am. Total sleep time was 498.21 min. Participants averaged 477.64 min in sedentary activity, 297.29 min in light activity, 150.66 min in moderate activity, and 18.05 min in vigorous activity. Adjusted models suggest that high school students engage in significantly more sedentary activity, and significantly less light, moderate, and vigorous activity compared to those in middle and elementary school. All students engaged in less moderate and vigorous activity on the weekend compared to the weekday. Data suggest that as children age they become more sedentary. Future studies should focus on increasing daily PA in high school students while considering other obesogenic factors.

## 1. Introduction

Alaska Native people in Anaktuvuk Pass have historically had a very active and healthy lifestyle that is related their subsistence lifestyle. However, after colonization they began experiencing important cardio-metabolic health disparities [1]. Changes in culture and lifestyle were particularly recent and rapid for Alaska Native people, with deleterious consequences [2,3]. One of the authors (DM) shares some of this history in the community where the study took place.

“Anaktuvuk Pass was settled in 1949. It is said there were 3 reasons why the seven families chose this particular valley. The Brooks Range mountains provide numerous streams of melting glacial water. The willows were once large and plentiful for firewood. The caribou follow their ancestral trails in the valley each spring and fall during their migration to or from the winds of the North Slope.

We are located in the north central area of the Brooks Range. The closest town with a hospital, store, and restaurant is 250 air miles south. To the north, the closest town is approximately 250 air miles across the Beaufort Sea. Our home is in the mountains north of the arctic circle 2000 feet above sea level. Our population is approximately 300 people, with the majority Inupiaq. More specifically, Nunamiut or the people of the land.

The Nunamiut have been said to be the last nomadic people of North America. My father was part of these last nomadic Indigenous people. He passed away when I was a child but his hunting partner/best friend has shared some stories of their travels with me.

They would walk miles per day looking for caribou. Caribou has always been our main sustenance. Snaring rabbits, squirrels, or ptarmigan on their journeys would provide much needed food for these long walks searching for caribou. Looking for food in the Brooks Range mountains can be difficult. They had to traverse the rough terrain of tundra, foothills, and mountain peaks that can reach thousands of feet high. The tundra consists of tussocks with potholes that can be several feet deep, holding water that does not move, infested with mosquito larvae. In the summer, walking was avoided in the lower altitude areas as the mosquito infestation would be terrible. The tundra on the foothills of the mountains are full of rocks and boulders. Maneuvering through this landscape can be similar to maneuvering an obstacle course, always cautious not to get your foot or leg stuck in a crevice while jumping or hopping from boulder to boulder, which can severely damage your body. The less challenging tundra is flat with small rocks scattered around the dwarfed shrubbery created by lack of growth due to the Arctic climate. These more merciless areas are located on flat spots on the top of a foothill located at the bottom of the mountains. The amount of physical work that is needed for living a subsistence lifestyle, the Nunamiut hunters have been and continue to be in very good physical condition. My father and his paanaq (partner)—specifically hunting partner in this context—roamed the Brooks Range always seeking food. Dogs were utilized as they traveled long distances through this rough terrain. My father and his paanaq harnessed dog packs onto their dogs which carried supplies and the dismembered bodies of caribou if they were lucky to harvest any. The dog packs were constructed out of caribou hides, hand-tanned, and sewn together with the split, dried sinew of the caribou. One caribou skin dog pack is preserved at the Simon Paneak Memorial Museum in Anaktuvuk Pass and was made by my father.

My cousins and I were told as children that dogs would always help us if we took good care of them. There are stories of Simon Paneak (whom the museum is named after) who sat and picked the dried feces from his dogs behind because he wanted his dogs be clean. Up until the mid-1980s when the Gates of the Arctic National Park and Preserve bordered three sides of Anaktuvuk Pass, the Nunamiut used dogs for pulling sleds in the winter and pack in the summer. When the park was created, the Nunamiut were no longer allowed to feed their dogs solely caribou meat. There is no road into Anaktuvuk Pass so all store bought supplies have to be brought in by bush plane. A 50 pound (22.7 kg) bag of dog food may cost $60 but when you add on the $1 per pound freight charge, your 50 pound bag of dog food now costs $110. I have two dogs in Anaktuvuk Pass that consume about 150 pounds (68 kg) of dog food a month. This is $330 worth of dog food a month for two dogs.”

**Figure 1.**
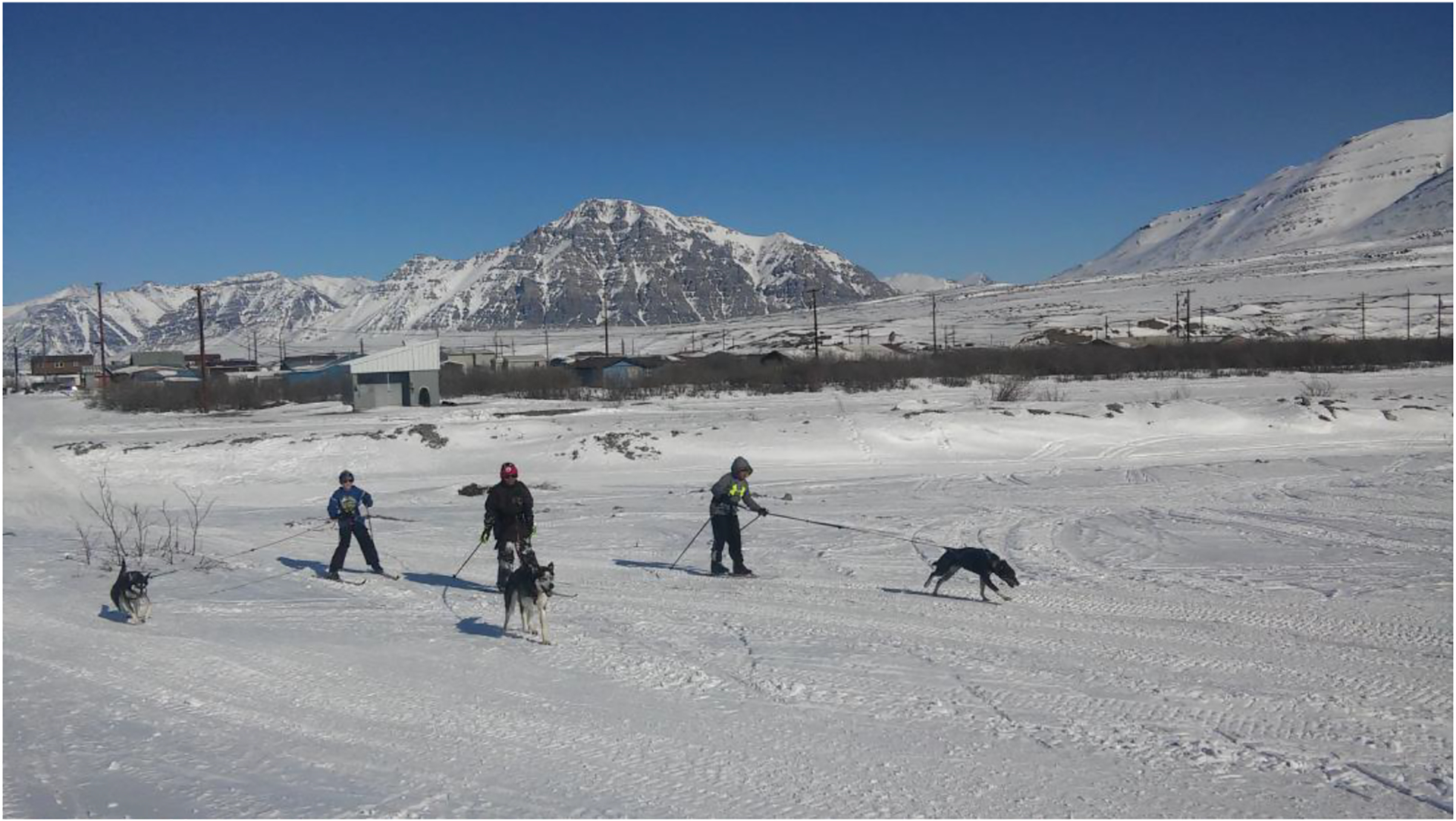
Youth from Anaktuvuk Pass participating in the skijoring program. In this arctic environment, April temperatures can be as low as −20 °F (−30 °C) and the closest town is 250 miles (400 km) south with no road or navigable waterway. Copyright Jacques Philip, 2017.

Explorer Helge Ingstad, who spent a winter with the Nunamiut tribe in 1949, noted that children were very healthy and happy, and hunters had remarkable physical abilities [4]. Many artifacts of the Nunamiut ancestral lifestyle are preserved in the Community of Anaktuvuk Pass Museum. Contemporary residents of Anaktuvuk Pass likely do not have the same level of physical activity (PA) as their predecessors, but until now no data were available to quantify this. This study is part of a broader effort to promote PA among youth in Alaska Native communities by developing the practice of cross-country skiing and incorporating the use of sled dogs (skijoring). This strategy may have additional mental health benefits, while minimizing the financial burden compared to sledding activities that would require more animals. The picture in Figure 1 illustrates the environment in which this study took place. It is important to note that sunlight in Anaktuvuk Pass varies between two hours at the heart of winter to 24 h a day at the peak of the summer. During data collection in April, sunrise occurred at 5:50 am and sunset at 10:20 pm. Because of low sunlight exposure and a nutritional transition, Alaska Native children exhibit high rates of vitamin D deficiency [5].

PA is a crucial modifiable behavior to enhance health. This is important to understand in American Indian and Alaska Native (AIAN) communities due to the alarmingly high rates of obesity [6–8] and the subsequent high risk of chronic disease [9–11]. In contrast, individuals engaging in adequate daily PA have profound health benefits that have been well established [12,13]. Unfortunately, most individuals do not obtain the recommended 60 min of PA per day [14]. A systematic review of the literature to determine a report card on how well the United States is doing with PA assigned a failing grade for overall PA levels in children and youth attaining 60 min or more of moderate-to-vigorous physical activity (MVPA) [15]. Other studies report that children spend 70% of their time in sedentary activity [16]. Accordingly, little work has been undertaken to objectively measure PA in AIAN children. A systematic review of PA in American Indian (AI) youth reports only four studies that objectively measure PA [17]. One study does not report outcome data [18], and two studies report outcome data in vector magnitude per hour, which is not comparable [19,20]. Other works undertaken with AI youth assessing objective measures do not report cross-sectional PA patterns [21], and one study with urban AI youth reports an average of 178.80 min of MVPA per day [22]. Moreover, pedometer and accelerometer data in Yup’ik adults report an average of 7531 steps per day [23] and an average of 201.90 min of MVPA/day [24], respectively. The sparse amount of work supports the need to better understand PA patterns in AIAN youth, especially Nunamiut in Anaktuvuk Pass.

PA patterns have been shown to dramatically shift from the weekday to the weekend. In the general population, studies report that youth engage in less PA on the weekend compared to the weekday, with decreases ranging from 6.07 min to as high as 18.00 min [16,25–27]. Sedentary activity has also been reported to significantly shift from weekday to weekend [26]. Studies show that children obtain the most MVPA during weekday school time [28]. However, little is known about PA patterns in AIAN youth. Brusseau et al. [29] collected PA with pedometry in 5th–6th grade children in a southwestern AI community and found average weekday and weekend steps at 11,891 and 7370, with an average difference of 4521 steps. Grant et al. [22] conducted a cross-sectional study in urban AI youth with accelerometry and found a significant decrease in light, moderate, vigorous and MVPA, and a significant increase in sedentary activity on the weekend compared to the weekday. These studies demonstrate the variability in PA from the weekday to weekend and the sparse understanding about PA patterns in AIAN youth.

Arguably, sleep research is still in its infancy and sleep patterns in youth are vaguely understood. Understanding sleep patterns is important as children aged 6 years to 17 years report high levels of inadequate sleep [30]. This is a particular concern for AIAN youth, as previous work suggests that minority youth sleep less than Caucasian youth [31]. Studies in youth report sleep duration ranges between 7.30 h and 9.40 h, bedtime ranges between 10:45 pm and 11:18 pm, and waketime ranges between 6:45 am and 7:51 am, respectively [22,32–35]. Understanding youth sleep patterns is critical in order to maintain regular waketime and nightly sleep duration.

Sleep changes mirror PA in terms of shifts from weekday to weekend. Several studies report increased sleep duration on the weekend compared to the weekday, with shifts ranging from 12.00 min to as much as an hour [26,36–40]. Another study assessing children aged 12–18 discovered weekend sleep shifts of 111.00 min, just shy of two hours [41]. Moreover, a study with youth of mean age 14 years reported that 66% of the participants had a weekday to weekend shift of sleep > 2 h in favor of the weekend [35]. According to the literature, there is one study that reports weekday to weekend shifts in sleep with AIAN youth in this age group. Grant et al. [22] conducted a study with urban AI children in the 6th–8th grade and found a weekend increase of 42.50 min. These data in youth underscore the variability in sleep and demonstrate that weekday to weekend sleep is not maintained.

The Center for Alaska Native Health Research (CANHR) study has carried out work with Alaska Native populations focused on obesity genetics, community nutrition and PA, and cultural–behavioral health measurement and development [42]. However, much of this work reports on risk factors and the prevalence of metabolic syndrome in Southwest Alaskan tribes [43,44]. This study builds upon the existing work that has been carried out, with a specific focus on sleep and PA in Nunamiut youth. Therefore, the purpose of this study is to describe PA, sleep, height and weight variables in elementary through high school youth and children living in Anaktuvuk Pass in Alaska.

## 2. Materials and Methods

### 2.1. Study Design and Participants

This work was a collaboration between CANHR at the University of Alaska Fairbanks (UAF), the community of Anaktuvuk Pass, the Nunamiut School, and the Alaska Nordic ski program. It was guided by community-based participatory research principles; in particular, the project leader obtained permission from the tribal council to submit the proposal and established a community steering committee charged with guiding the research and insuring its cultural relevance. The long-term goal of the project was to establish dogjoring activities (a dog pulling a skier or hiker) as a supplement to an existing cross-country ski program provided by an outside entity to increase youth motivation to engage in PA. The assumption is that Nunamiut children are naturally attracted to interact with dogs, with the added bonus that Anaktuvuk Pass has a rich history of using dogs for packing and sledding, thus increasing the cultural relevance of the program. The study presented here is a cross-sectional pilot assessing PA and sleep in Nunamiut children. All procedures were approved by the UAF Institutional Review Board (IRB), under protocol # 1089068.

### 2.2. Instruments and Variables

Data collection occurred during one week in April, as the Alaska Nordic ski program, including dogjoring activities, was ongoing in the community. The research team, composed of the project leader and three ski/skijoring coaches, traveled to Anaktuvuk Pass for a week. All elementary to high school students aged 12–18 were invited, and 38 participated. The ski program was integrated in physical education classes during winter and spring. In addition, during the week of data collection, the dogjoring activities supplemented the skiing activities, allowing each student to participate in one hour of daily skiing/skijoring during the school day and with the option of one to two additional hours after school. Other after school activities, such as basketball, were also available to students. Overall, the skijoring activities did not represent an unusual amount of PA for the youth as they typically participate in various afterschool activities. Each youth participant signed an assent form and provided parental consent, which was facilitated by a high school student (i.e., research assistant) who received IRB training. Students were called in small groups to a station in the school, and were asked to complete a short demographic questionnaire, participate in anthropometric measurements, and an accelerometer was administered. Each student received a USD 25 gift card for Amazon upon enrollment, and another USD 25 gift card for Amazon when they returned the accelerometer a week later.

#### 2.2.1. Physical Activity and Sleep

PA and sleep were assessed by the ActiGraph GT3X+ accelerometer (Actigraph Pen-sacola, FL, USA), a valid measure of activity energy expenditure in youth [45]. Accelerometers were fitted on the non-dominant wrist. The devices’ frequency was set at 90 Hz, which allows for maximum data density. Participants were instructed to wear the devices 24 h/day for seven days starting on Monday. Weekend PA periods were defined as occurring Saturday or Sunday and weekend sleep periods include Friday and Saturday night. For PA, a participant must have three valid weekdays and one valid weekend day, with a day having a minimum of 480.00 min (eight hours) of wear time. For sleep, a participant must have three weekday and one weekend sleep period.

The PA cutpoints were based on counts per five second epochs on the Y axis: sedentary 0.00 to 35.00, light 36.00 to 360.00, moderate 361.00 to 1129.00, and vigorous 1130.00 and above [46]. Sleep measures are: in bedtime (IBT) and out of bedtime (OBT), as identified by automatic sleep detection in Actilife, time in bed (TIB), defined as the difference between OBT and IBT, and sleep efficiency as the number of sleep minutes divided by TIB [47,48].

#### 2.2.2. Body Composition

Height was measured to the nearest 0.10cm with a portable stadiometer (Perspective Enterprises, PE-AIM-101; Portage, MI, USA), weight was measured to the nearest 0.10 kg with a portable scale (Seca Model 876; Chino, CA, USA), and waist circumference was measured to the nearest 0.10 cm with a plastic tape (Seca Model 201; Chino, CA, USA). All measurements were recorded three times for each participant. Measures were repeated if they were not within 0.20 units (e.g., 0.20 kg for weight). BMI percentiles and z-scores (BMI-Z) were calculated in SAS using the Centers for Disease Control and Prevention calculator [49]. The analysis was unable to compute BMIZ and BMI percentiles for one participant because he was over 20 years old, which is above the limit for those measures.

#### 2.2.3. Demographics

Participants completed an eight-item demographic survey that assessed age, grade, gender, race and ethnicity (i.e., Alaska Native). Traditional and Western lifestyle were assessed with three options: “Not at all”, “Some” and “A lot”. These variables measured how much an individual identified with living a traditional Nunamiut lifestyle or identified with living a Western colonial lifestyle.

### 2.3. Procedure

ActiLife V6.13.40 software was used to download accelerometer raw data and generate activity counts on three axis, with 60.00 s epochs for sleep and five second epochs for PA. Sleep and PA data are traditionally analyzed in 60.00 s epochs; however, recent work showed that five second epochs are better at detecting short bursts of vigorous activity in youth [46]. A customized version of the Choi algorithm [50] was used to detect non-wear periods with the following parameters: minimum length: 60.00 min (the minimum length of time for consecutive zero counts to be considered a non-wear time interval), small window length: 30.00 min (the minimum length of up/down-stream time window for consecutive zero counts required before and after the artifactual movement interval to be considered a non-wear time interval), and spike tolerance: two minutes (the maximum length of time interval allowed for the artifactual non-zero counts during a non-wear time interval). Ignore wear periods were defined as less than 30.00 min.

The Sadeh algorithm on one minute epoch data was used to score sleep [48,51] as well as the automatic sleep period detection feature in Actilife, using a custom Tudor-Locke [52] algorithm with the following parameters: minimum sleep period of 160.00 min, waketime definition of 30.00 min of waketime, bedtime definition of five minutes sleep time, max time between bedtime and waketime of 1440.00 min, and 15.00 min of non-zero epochs. Due to the algorithm requiring several minutes of ‘sleep’ epochs at the start of a sleep period, the latency will be zero for any sleep periods that are found. Manual adjustments after automatic sleep detection were made to remove nap periods and join sleep periods that occurred the same night. After all the pre-processed accelerometry datasets were computed, the data were then imported into R and merged with the demographic and measurement data for analysis.

### 2.4. Data Analysis

Summary statistics and inference were computed in R v. 4.0. Summary statistics of the time of day were computed using the circular package v. 0.4–93. ANOVA tests were used to compare numerical differences, e.g., MVPA and a time specific ANOVA from the circular package (aov.circular function) was used to test time differences. Linear mixed models were constructed to test differences in PA with covariates and to take advantage of the within and across participant variability (each participant had multiple days). Diagnostics plots of linear mixed models showed that the only assumption not met was a deviation from normality. Therefore, Linear Quantile Mixed Models (LQMMs) were fit instead, using the lqmm R package v. 1.5.2.

## 3. Results

### 3.1. Demographics and Body Composition

A total of 38 children participated in this study (n = 27 males (71.05%); Table 1). Grade breakdown consisted of 17 (44.74%) in elementary school, nine (23.68%) in middle school, and 12 (31.58%) in high school, with a mean age of 12.82 ± 2.80. Body composition found that four (10.53%) were overweight and seven (18.42%) obese.

### 3.2. Sleep Patterns

Participants’ average bedtime was 00:15 am and waketime 08:23 am, with an average total sleep time of 498.21 min (Table 2). Shifts were observed from weekday to weekend. For instance, weekday bedtime was 00:04 am and weekend bedtime 00:43 am (Δ 39 min; *p*-value = 0.061), and weekday waketime was 08:00 am and weekend waketime 09:25 am (Δ 85 min; *p*-value ≤ 0.001). Weekday total sleep time was 480.46 min and weekend total sleep time was 541.44 min (Δ 60.98 min; *p*-value ≤ 0.01).

### 3.3. Physical Activity Patterns

Participants on average engaged in 477.64 min sedentary activity, 297.29 min light activity, 150.66 min moderate activity, 18.05 min vigorous activity, and 168.71 min MVPA (Table 3). Shifts were observed from weekday to weekend. For instance, sedentary activity was 474.43 min during weekday and 485.66 min during weekend (Δ 11.23 min; *p*-value = 0.58), light activity was 300.67 min during weekday and 288.84 min during weekend (Δ 11.83 min; *p*-value = 0.298), moderate activity was 157.46 min during weekday and 133.66 min during weekend (Δ 23.80 min; *p*-value = 0.08), vigorous activity was 21.30 min during weekday and 9.92 min during weekend (Δ 11.38 min; *p*-value ≤ 0.001), and MVPA was 178.77 min during weekday and 143.58 min during weekend (Δ 35.19 min; *p*-value ≤ 0.001).

### 3.4. Quantile Linear Regression Models

Adjusted PA models show that high school youth engaged in significantly more sedentary activity (β = 122.55 min; *p*-value ≤ 0.001), and significantly less light (β = −54.27 min; *p*-value ≤ 0.01), moderate (β= *−*67.50 min; *p*-value ≤ 0.001), and vigorous PA (β = *−*10.28 min; *p*-value ≤ 0.001), compared to middle school and elementary school youth and children (Table 4). Results also show that all participants engaged in significantly less moderate (β = *−*32.15 min; *p*-value ≤ 0.001) and vigorous PA (β = *−*9.63 min; *p*-value ≤ 0.001) on the weekend compared to the weekday. Boys engaged in slightly more PA and less sedentary time than girls; however, none of the differences were statistically significant.

## 4. Discussion

The purpose of this study was to describe PA and sleep patterns in school-aged youth and children living in an isolated Alaska Native community in Anaktuvuk Pass. Results show that high school participants engage in significantly more sedentary activity and significantly less light, moderate, and vigorous activity compared to all other participants. In addition, all participants had shifts in activity patterns from weekday to weekend, such that moderate activity and vigorous activity were significantly less on the weekend. This held true for sleep, which showed shifts in bedtime, waketime, and total sleep time with more minutes during the weekend compared to the weekday.

The PA patterns observed in this study are consistently documented in the literature, and it appears that as children age to youth, they move less. For example, studies show that youth primarily engage in PA during school [53] and engage in more sedentary activity, such as increased screen time, outside of school [54,55]. This trend is also observed in longitudinal studies, with a decrease in PA and increase in screen time as children age [56–59]. This is an important consideration as research has shown a relationship between decreased PA and increase in BMI [60], which worsens as youth age in to adulthood [61]. These trends have important implications for the community of Nunamiut. First, the youth live in an isolated, underserved community with limited PA opportunities. Second, Nunamiut derive from generations of hunters and gatherers that possessed immense physical abilities attributed to their subsistence lifestyle. Colonialism has severely disrupted that way of life and caused deleterious effects on the PA of Nunamiut. Finally, this work is important to highlight the needs that exist in this community, specific to finding strategies to increase PA in this population.

This sample of Nunamiut youth and children engaged in significantly less moderate and vigorous activity on the weekend compared to the weekday. This study aligns with much of the literature assessing PA levels in the general population. Several studies report shifts ranging from 6.70 min to 18.00 min in terms of less MVPA on the weekend compared to weekday [16,25–27]. This trend also holds true for sedentary activity, where others report that sedentary activity increases on the weekend compared to the weekday [26]. These patterns were also observed in urban AI youth, where sedentary activity increased and MVPA decreased on the weekend compared to the weekday [22]. It appears that youth are obtaining the majority of their PA on the weekday during school. For instance, fewer youth achieve ≥60 min of MVPA on weekends compared to weekdays (46% vs. 22%;*p* ≤ 0.001), which equates to fourfold higher odds of not obtaining ≥60 min of MVPA on the weekend [16]. Because of the small sample size, the analysis was unable to assess gender differences, which is an important consideration. Studies report that boys not only engage in more MVPA than girls, but demonstrate less of a shift from the weekday to weekend than girls [28]. This has implications for researchers working to design PA interventions, suggesting tailoring the design to ensure girls are not only increasing activity levels, but also mitigating the PA decline on the weekend. It is important to mitigate weekday to weekend shifts because a decrease in MVPA and increase in sedentary activity show that youth have higher odds of obesity [26].

The overall average MVPA of 168 min/day is largely above the current recommendation of 60 min/day [14], indicating that youth and children from Anaktuvuk Pass may have retained some of the high activity behavior of their elders. A high level of fitness was confirmed by an informal qualitative assessment administered by the ski coaches (data not shown). Directly comparing activity levels between studies is challenging because of the differences in accelerometers, epochs, cut-points, and treatment of missing data. However, a study by Kim et al. [62] used a similar methodology on a sample of US youth and children of comparable ages and reported an average MVPA of 106.90 ± 1.80 min/day. In contrast, 40% of the participants in this study are overweight or obese compared to approximately 20% in the general population, highlighting the importance of other obesogenic factors. An important consideration is the introduction of sugary foods and drinks, while Nunamiut had been eating mostly a protein/fat diet for generations, which suggests that they have not adapted to sugary diets. In addition, the stress imposed by colonization has been proposed as contributing to obesity in Alaska Native people [23].

The sample of Nunamiut youth and children in this study demonstrated shifts amongst different sleep variables from the weekday to weekend. Specifically, weekday bedtime was 00:04 am and weekend bedtime was 00:43 am, a bedtime shift of 39.00 min later on the weekend. The same trends were observed for waketime, such that weekday waketime was 8:00 am and weekend waketime was 9:25 am, 1 h 25 min of sleeping in on the weekend. Moreover, total sleep time was 60.50 min more on the weekend compared to weekday. The literature shows similar patterns in shifts from weekday to weekend in total sleep time of 14.40 min to 111.10 min [22,36,39–41]. Weekday to weekend shifts in sleep are concerning since studies show that the quality of sleep is severely interrupted. For instance, 67.40% of youth report getting less than seven hours of sleep and 41.10% report that they do not get enough sleep [41]. One study reports that 63.20% of youth do not meet the recommended amount of weekday sleep and 50.60% do not meet the recommended amount of weekend sleep [38]. Sleep satisfaction is also inadequate according to self-report, where 74% of youth report that their sleep satisfaction is moderate or unsatisfactory [41] and other studies report that two-thirds of youth get < 8 h sleep per night [35]. A longitudinal study of 343 youth in 10th–12th grade reports a linear decline in weekday sleep of 0.35 h and weekend sleep of 0.45 h from the 10th grade to three years post high school [40]. This suggests that not only are there weekday to weekend shifts in sleep, but children are getting less sleep overall as they age. Perhaps weekend sleep shifts are attributed to later bedtimes. A study reports that school-aged children with a late bedtime have longer sleep onset latency and also sleep one hour less at night compared to children with an early bedtime [34]. In addition, studies show that a greater shift towards more total sleep time on the weekend is associated with greater eating in absence of hunger, which contributes to weight gain and obesity [39]. The literature clearly supports the need for anchoring a regular morning waketime, as this has been shown to be the best way to manage behavioral sleep problems [63]. Future work should also assess nightly screen time [64–68] and sleep hygiene practices [69–72] when assessing sleep duration.

### Strengths and Limitations

The strength of this study is the objective measurement of sleep and PA collected from an otherwise unknown, isolated, and underserved population. The research team successfully recruited 38 students to participate in this ongoing work to develop the dogjoring program. Conducting a study with this isolated Nunamiut community is a strength in and of itself. Not only does this study show trends that suggest the Nunamiut have adopted a western lifestyle, but this is perhaps more deleterious since there are fewer community resources (e.g., health care systems and health care providers) to address these issues. Although the research team was able to recruit 38 participants, this small sample size limits sensitivity in data analysis. In addition, the cross-sectional analysis provides only descriptive results. Finally, the analysis included overall trends in the data, and the contrast between high levels of PA and a high obesity prevalence suggests that interventions aimed at reducing obesity should address other obesogenic factors in addition to promoting PA. Future work needs to be undertaken with a larger sample size and assessing gender differences in sleep and PA. Sleep assessments should include nightly screen time and sleep hygiene. In spite of these limitations, future work will build upon this work to gain a deeper understanding of sleep and PA in this population.

## 5. Conclusions

This study presents data on sleep, PA, and demographic information from 38 Nunamiut youth and children living in Anaktuvuk Pass in Alaska. Results show that high school youth engage in more sedentary activity, and less light, moderate, and vigorous PA, compared to middle school and elementary school children. This suggests that as children age into youth they move less. All participants demonstrated shifts in favor of less PA, more sedentary activity, and variable sleep patterns on the weekend compared to the weekday. Finally, approximately 11 (40.70%) of the participants were either overweight or obese. Future work should consider the time of year the research is carried out. This is important, especially in the fall, when Nunamiut high school youth are engaged in hunting, and may provide a more realistic look at PA trends in this population. In addition, differences in gender should be examined in order to determine if males and females have different physical activity and sleep needs.

## Figures and Tables

**Table 1.** Demographic and anthropometric statistics (n = 38).

Variables	N (%) or Mean ± sd
Age	
[9–11]	14 (36.84)
[12–15]	17 (44.74)
[16–20]	7 (18.42)
Gender	
Male	27 (71.05)
Female	11 (28.95)
Grade	
Elementary [4–6]	17 (44.74)
Middle school [7,8]	9 (23.68)
High school [9–12]	12 (31.58)
Race/Ethnicity	
Inupiaq	34 (89.47)
White	4 (10.53)
Traditional lifestyle	
Not at all	1 (2.63)
Some	24 (63.16)
A lot	13 (34.21)
Western lifestyle	
Not at all	3 (7.89)
Some	21 (55.26)
A lot	14 (36.84)
Combined lifestyle score [2–6]	
3	1 (2.63)
4	16 (42.11)
5	18 (47.4)
6	3 (7.89)
BMI percentile^1^	
Normal weight	26 (68.42)
Overweight	4 (10.53)
Obese	7 (18.42)
Missing	1 (2.63)
BMIZ	
Elementary [4–6]	0.64 ± 1.01
Middle school [7,8]	0.58 ± 0.68
High school [9–12]	1.02 ± 1.16
All	0.74 ± 0.98

1 BMI percentiles: 0–5th = underweight; 5th–85th = normal weight; 85th–95th = overweight; ≥95th = obese.

**Table 2.** Descriptive statistics of sleep (n = 33).

Variables	Weekday (N or Mean ± sd)	Weekend (N or Mean ± sd)	All (N or Mean ± sd)	*p*-Value
Number of sleep periods (n)	151	62	213	
Average in bed time (hh:mm)	00:04 AM ± 00:35	00:43 AM ± 00:38	00:15 AM ± 00:36	0.061
Average out of bed time (hh:mm)	08:00 AM ± 00:23	09:25 AM ± 00:34	08:23 AM ± 00:28	≤0.001
Average time in bed (min)	480.46 ± 139.23	541.44 ± 183.09	498.21 ± 155.35	0.009
Average sleep efficiency (%)	86.24 ± 7.12	85.34 ± 8.13	85.98 ± 7.42	0.424
Average wake after sleep onset (min)	66.57 ± 42.96	80.50 ± 55.73	70.62 ± 47.33	0.051
Average period sleep total time (min)	480.19 ± 139.28	540.69 ± 183.19	497.80 ± 155.4	0.010
Average number of awakenings (n)	20.63 ± 8.29	23.79 ± 12.14	21.55 ± 9.65	0.030
Average length of awakenings (min)	3.18 ± 1.4	3.40 ± 1.78	3.25 ± 1.50	0.345
Average period activity count (n)	30,805.54 ± 24,175.20	35,319.94 ± 27,155.57	32,119.59 ± 25,098.4	0.234
Average movement index (MI)^1^	11.89 ± 4.24	12.79 ± 5.56	12.15 ± 4.67	0.203
Average fragmentation index (FI)^2^	10.46 ± 8.06	10.60 ± 7.79	10.50 ± 7.96	0.904
Average sleep fragmentation index (SFI)^3^	22.34 ± 10.4	23.4 ± 11.50	22.65 ± 10.71	0.519

1 MI = percentage of epochs with y-axis counts greater than zero in the sleep period.

2 FI = percentage of one minute periods of sleep vs. all periods of sleep during the sleep period.

3 SFI = sum of MI and FI.

**Table 3.** Descriptive statistics of physical activity (n = 31).

Variables	Weekday (Mean ± sd or %)	Weekend (Mean ± sd or %)	All (Mean ± sd or %)	*p*-Value
Activity cut-points				
Average sedentary time/day (min)	474.43 (129.51)	485.66 (140.04)	477.64 (132.36)	0.580
Average proportion of sedentary time/day (%)	49.66	52.91	50.56	
Average light activity time/day (min)	300.67 (60.00)	288.84 (101.85)	297.29 (74.32)	0.298
Average proportion of light activity time/day (%)	31.47	31.47	31.47	
Average moderate activity time/day (min)	157.46 (52.95)	133.66 (69.52)	150.66 (58.99)	0.008
Average proportion of moderate activity time/day (%)	16.48	14.56	15.95	
Average vigorous activity time/day (min)	21.30 (16.71)	9.92 (12.95)	18.05 (16.52)	≤0.001
Average proportion of vigorous activity time/day (%)	2.23	1.08	1.91	
Average MVPA time/day (min)	178.77 (64.23)	143.58 (78.00)	168.71 (70.09)	≤0.001
Average proportion of MVPA time/day (%)	18.71	15.64	17.86	

Note: Numbers are adjusted for wear time (missing data).

**Table 4.** Quantile linear mixed regression models of different PA activities adjusted by gender, grade, and weekend.

	Average Sedentary Time/Day (min)		Average Light Time/Day (min)		Average Moderate Time/Day (min)		Average Vigorous Time/Day (min)	
	β	*p*-Value	β	*p*-Value	β	*p*-Value	β	*p*-Value
Intercept	425.62	≤0.001	314.46	≤0.001	200.64	≤0.001	21.24	≤0.001
Female	35.27	0.39	−4.40	0.82	−20.04	0.14	−1.90	0.52
High School *	122.55	≤0.001	−54.27	≤0.01	−67.50	≤0.001	−10.28	≤0.001
Weekend	12.70	0.43	−17.60	0.27	−32.15	≤0.001	−9.63	≤0.001

* Grade 9–12 vs. all other participants

## Data Availability

The data presented in this manuscript were obtained through a NIH-funded research collaboration with the Nunamiut people of Anaktuvuk Pass in the Brooks Range mountains of Alaska. They were generated through the actions of the research team and in partnership with the people of Anaktuvuk Pass. The community has approved the publication and dissemination of the data in the format provided in this manuscript. The primary dataset from our study is owned by the people of Anaktuvuk Pass, in recognition of tribal sovereign authority stipulated by the government of the United States. Requests for access to the primary data can be made to the Naqsragmiut tribal leadership. While we cannot speak for them, we believe that all requests would be given due consideration. Such requests can be directed to Jacques Philip.

## References

[1] Stoner L; Matheson AG; Perry LG; Williams MA; McManus A Principles and strategies for improving the prevention of cardio-metabolic diseases in indigenous populations: An international Delphi study. Prev. Med 2017, 96, 106–112. 28057511 10.1016/j.ypmed.2016.12.050

[2] de Schweinitz PA; Wojcicki JM First Nations approaches to childhood obesity: Healthy lifestyles in Canada compared with alternatives for Alaska Native communities. Children 2017, 4, 38. 28492517 10.3390/children4050038 PMC5447996

[3] Espey DK; Jim MA; Cobb N; Bartholomew M; Becker T; Haverkamp D; Plescia M Leading causes of death and all-cause mortality in American Indians and Alaska Natives. Am. J. Public Health 2014, 104, 303–311. 10.2105/AJPH.2013.301798 PMC4035872 24754554

[4] Ingstad H; Spearman GR Nunamiut: Among Alaska’s Inland Eskimos Special commemorative; Countryman Press: Woodstock, VT, USA, 2006.

[5] Singleton R; Lescher R; Gessner BD; Benson M; Bulkow L; Rosenfeld J; Thomas T; Holman RC; Haberling D; Bruce M; Rickets and vitamin D deficiency in Alaska native children. J. Pediatr. Endocrinol. Metab 2015, 28, 815–823. 25741788 10.1515/jpem-2014-0446 PMC4678029

[6] Dennison ME; Sisson SB; Lora K; Stephens LD; Copeland KC; Caudillo C Assessment of Body Mass Index, Sugar Sweetened Beverage Intake and Time Spent in Physical Activity of American Indian Children in Oklahoma. J. Community Health 2015, 40, 808–814. 25750107 10.1007/s10900-015-0004-6

[7] Noonan CW; Brown BD; Bentley B; Conway K; Corcoran M; Fourstar K; Freide P; Hemlock B; Wagner S; Wilson T Variability in childhood asthma and body mass index across northern plains American Indian communities. J. Asthma 2010, 47, 496–500. 20560824 10.3109/02770901003759436

[8] Pan L; McGuire LC; Blanck HM; May-Murriel AL; Grummer-Strawn LM Racial/ethnic differences in obesity trends among young low-income children. Am. J. Prev. Med 2015, 48, 570–574. 25891056 10.1016/j.amepre.2014.11.009 PMC4582763

[9] Hu FB; Manson JE; Stampfer MJ; Colditz G; Liu S; Solomon CG; Willett WC Diet, lifestyle and the risk of type-2 diabetes mellitus in women. N. Engl. J. Med 2001, 345, 790–797. 11556298 10.1056/NEJMoa010492

[10] Hutchinson RN; Shin S Systematic review of health disparities for cardiovascular diseases and associated factors among American Indian and Alaska Native populations. PLoS ONE 2014, 9, e80973. 24454685 10.1371/journal.pone.0080973 PMC3893081

[11] Sinclair KA; Bogart A; Buchwald D; Henderson JA The prevalence of metabolic syndrome and associated risk factors in northern plains and southwest American Indians. Diabetes Care 2011, 34, 118–120. 20864516 10.2337/dc10-0221 PMC3005451

[12] Council on Sports Medicine and Fitness and Council on School Health. Active healthy living: Prevention of childhood obesity through increased physical activity. Pediatrics 2006, 117, 1834–1842. 16651347 10.1542/peds.2006-0472

[13] Bouchard C; Shephard RJ; Stephens T Exercise, Fitness, and Health: International Proceedings and Consensus Statement; Human Kinetics: Champaign, IL, USA, 1994.

[14] Foulds HJ; Warburton DE; Bredin SS A systematic review of physical activity levels in Native American populations in Canada and the United States in the last 50 years. Obes. Rev 2013, 14, 593–603. 23577646 10.1111/obr.12032

[15] Dentro KN; Beals K; Crouter SE; Eisenmann JC; McKenzie TL; Pate RR; Saelens BE; Sisson SB; Spruijt-Metz D; Sothern MS; Results from the United States’ 2014 report card on physical activity for children and youth. J. Phys. Act. Health 2014, 11, S105–S112. 25426905 10.1123/jpah.2014-0184

[16] Comte M; Hobin E; Majumdar SR; Plotnikoff RC; Ball GD; McGavock J; MIPASS and Healthy Hearts Investigators Teams. Patterns of weekday and weekend physical activity in youth in 2 Canadian provinces. Appl. Physiol. Nutr. Metab 2013, 38, 115–119. 23438220 10.1139/apnm-2012-0100

[17] Fleischhacker S; Roberts E; Camplain R; Evenson KR; Gittelsohn J Promoting physical activity among Native American youth: A systematic review of the methodology and current evidence of physical activity interventions and community-wide initiatives. J. Racial Ethn. Health Disparities 2016, 3, 608–624. 27294756 10.1007/s40615-015-0180-1 PMC4911341

[18] Adams AK; LaRowe TL; Cronin KA; Prince RJ; Wubben DP; Parker T; Jobe JB The Healthy Children, Strong Families intervention: Design and community participation. J. Prim. Prev 2012, 33, 175–185. 22956296 10.1007/s10935-012-0275-y PMC3904366

[19] Going S; Thompson J; Cano S; Stewart D; Stone E; Harnack L; Hastings C; Norman J; Corbin C The effects of the Pathways Obesity Prevention Program on physical activity in American Indian children. Prev. Med 2003, 37, S62–S69. 14636810 10.1016/j.ypmed.2003.08.005

[20] Harvey-Berino J; Rourke J Obesity prevention in preschool Native-American children: A pilot study using home visiting. Obes. Res 2003, 11, 606–611. 12740449 10.1038/oby.2003.87

[21] Brown B; Noonan C; Harris KJ; Parker M; Gaskill S; Ricci C; Cobbs G; Gress S Developing and piloting the journey to Native youth health program in Northern Plains Indian communities. Diabetes Educ. 2013, 39, 109–118. 23150531 10.1177/0145721712465343

[22] Grant VM; Tomayko EJ; Kingfisher RD Sleep and Physical Activity Patterns in Urban American Indian Children. Am. J Health Behav 2020, 44, 67–75. 31783933 10.5993/AJHB.44.1.7 PMC7373171

[23] Bersamin A; Wolsko C; Luick BR; Boyer BB; Lardon C; Hopkins SE; Stern JS; Zidenberg-Cherr S Enculturation, perceived stress, and physical activity: Implications for metabolic risk among the Yup’ik–The Center for Alaska Native Health Research Study. Ethn. Health 2014, 19, 255–269. 23297688 10.1080/13557858.2012.758691 PMC3644025

[24] Philip J; Ryman TK; Hopkins SE; O’Brien DM; Bersamin A; Pomeroy J; Thummel KE; Austin MA; Boyer BB; Dombrowski K Bi-cultural dynamics for risk and protective factors for cardiometabolic health in an Alaska Native (Yup’ik) population. PLoS ONE 2017, 12, e0183451. 29091709 10.1371/journal.pone.0183451 PMC5665420

[25] Konharn K; Santos MP; Ribeiro JC Differences between weekday and weekend levels of moderate-to-vigorous physical activity in Thai adolescents. Asia Pac. J. Public Health 2015, 27, NP2157–NP2166. 23007484 10.1177/1010539512459946

[26] Li N; Zhao P; Diao C; Qiao Y; Katzmarzyk PT; Chaput JP; Fogelholm M; Kuriyan R; Kurpad A; Lambert EV; Joint associations between weekday and weekend physical activity or sedentary time and childhood obesity. Int. J. Obes 2019, 43, 691–700. 10.1038/s41366-019-0329-9 PMC6445763 30705394

[27] Ramirez-Rico E; Hilland TA; Foweather L; Fernández-Garcia E; Fairclough SJ Weekday and weekend patterns of physical activity and sedentary time among Liverpool and Madrid youth. Eur. J. Sport Sci 2014, 14, 287–293. 23984835 10.1080/17461391.2013.827242

[28] Aibar A; Bois JE; Zaragoza Casterad J; Generelo E; Paillard T; Fairclough S Weekday and weekend physical activity patterns of French and Spanish adolescents. Eur. J. Sport Sci 2014, 14, 500–509. 23978199 10.1080/17461391.2013.829127

[29] Brusseau TA; Kulinna PH; Tudor-Locke C; Ferry M Daily physical activity patterns of children living in an American Indian community. J. Phys. Act. Health 2013, 10, 48–53. 23324486 10.1123/jpah.10.1.48

[30] Hawkins SS; Takeuchi DT Social determinants of inadequate sleep in US children and adolescents. Public Health 2016, 138, 119–126. 27207726 10.1016/j.puhe.2016.03.036 PMC5012907

[31] Combs D; Goodwin JL; Quan SF; Morgan WJ; Parthasarathy S Longitudinal differences in sleep duration in Hispanic and Caucasian children. Sleep Med. 2016, 18, 61–66. 26299467 10.1016/j.sleep.2015.06.008 PMC4548806

[32] Chen G; Ratcliffe J; Olds T; Magarey A; Jones M; Leslie E BMI, health behaviors, and quality of life in children and adolescents: A school-based study. Pediatrics 2014, 133, e868–e874. 24590749 10.1542/peds.2013-0622

[33] Harrington SA Relationships of objectively measured physical activity and sleep with BMI and academic outcomes in 8-year-old children. Appl. Nurs. Res 2013, 26, 63–70. 23583266 10.1016/j.apnr.2013.02.001

[34] Mindell JA; Meltzer LJ; Carskadon MA; Chervin RD Developmental aspects of sleep hygiene: Findings from the 2004 National Sleep Foundation Sleep in America Poll. Sleep Med. 2009, 10, 771–779. 19285450 10.1016/j.sleep.2008.07.016

[35] Wing YK; Chan NY; Man Yu MW; Lam SP; Zhang J; Li SX; Kong APS; Li AM A school-based sleep education program for adolescents: A cluster randomized trial. Pediatrics 2015, 135, e635–e643. 25687152 10.1542/peds.2014-2419

[36] Hasler BP; Dahl RE; Holm SM; Jakubcak JL; Ryan ND; Silk JS; Phillips ML; Forbes EE Weekend–weekday advances in sleep timing are associated with altered reward-related brain function in healthy adolescents. Biol. Psychol 2012, 91, 334–341. 22960270 10.1016/j.biopsycho.2012.08.008 PMC3490026

[37] Hayes JF; Balantekin KN; Altman M; Wilfley DE; Taylor CB; Williams J Sleep patterns and quality are associated with severity of obesity and weight-related behaviors in adolescents with overweight and obesity. Child. Obes 2017, 14, 11–17. 28850274 10.1089/chi.2017.0148 PMC5743029

[38] James S; Chang AM; Buxton OM; Hale L Disparities in adolescent sleep health by sex and ethnoracial group. SSM Popul. Health 2020, 11, 100581. 32373706 10.1016/j.ssmph.2020.100581 PMC7191202

[39] LeMay-Russell S; Tanofsky-Kraff M; Schvey NA; Kelly NR; Shank LM Associations of weekday and weekend sleep with children’s reported eating in the absence of hunger. Nutrients 2019, 11, 1658. 31330788 10.3390/nu11071658 PMC6682878

[40] Park H; Chiang JJ; Irwin MR; Bower JE; McCreath H; Fuligni AJ Developmental trends in sleep during adolescents’ transition to young adulthood. Sleep Med. 2019, 60, 202–210. 31186213 10.1016/j.sleep.2019.04.007 PMC6650269

[41] Chung JH; Kim JB; Kim JH Effects of sleep duration and weekend catch-up sleep on falling injury in adolescents: A population-based study. Sleep Med. 2020, 68, 138–145. 32036286 10.1016/j.sleep.2019.12.005

[42] Boyer BB; Mohatt GV; Lardon C; Plaetke R; Luick BR; Hutchison SH; Antunez de Mayolo G; Ruppert E; Bersamin A Building a community-based participatory research center to investigate obesity and diabetes in Alaska Natives. Int. J. Circumpolar Health 2005, 64, 281–290. 16050322 10.3402/ijch.v64i3.18002

[43] Boyer BB; Mohatt GV; Plaetke R; Herron J; Stanhope KL; Stephensen C; Havel PJ Metabolic syndrome in Yup’ik Eskimos: The center for alaska native health research (CANHR) study. Obesity 2007, 15, 2535–2540. 18070741 10.1038/oby.2007.302

[44] Mohatt GV; Plaetke R; Klejka J; Luick B; Lardon C; Bersamin A; Hopkins S; Dondanville M; Herron J; Boyer B The Center for Alaska Native Health Research Study: A community-based participatory research study of obesity and chronic disease-related protective and risk factors. Int. J. Circumpolar Health 2007, 66, 8–18. 17451130 10.3402/ijch.v66i1.18219

[45] Cain KL; Sallis JF; Conway TL; Van Dyck D; Calhoon L Using accelerometers in youth physical activity studies: A review of methods. J. Phys. Act. Health 2013, 10, 437–450. 23620392 10.1123/jpah.10.3.437 PMC6331211

[46] Crouter SE; Flynn JI; Bassett DR Estimating physical activity in youth using a wrist accelerometer. Med. Sci. Sports Exerc 2015, 47, 944–951. 25207928 10.1249/MSS.0000000000000502 PMC4362848

[47] Acebo C; Sadeh A; Seifer R; Tzischinsky O; Wolfson AR; Hafer A; Carskadon MA Estimating sleep patterns with activity monitoring in children and adolescents: How many nights are necessary for reliable measures? Sleep 1999, 22, 95–103. 9989370 10.1093/sleep/22.1.95

[48] Sadeh A; Sharkey KM; Carskadon MA Activity-based sleep-wake identification: An empirical test of methodological issues. Sleep 1994, 17, 201–207. 7939118 10.1093/sleep/17.3.201

[49] Centers for Disease Control and Prevention. A SAS Program for the 2000 CDC Growth Charts (ages 0 to <20 years). Division of Nutrition, Physical Activity, and Obesity 2014. Available online: https://www.cdc.gov/nccdphp/dnpao/growthcharts/resources/sas.htm (accessed on 3 June 2022).

[50] Choi L; Liu Z; Matthews CE; Buchowski MS Validation of accelerometer wear and nonwear time classification algorithm. Med. Sci. Sports Exerc 2011, 43, 357–364. 20581716 10.1249/MSS.0b013e3181ed61a3 PMC3184184

[51] Quante M; Kaplan ER; Cailler M; Rueschman M; Wang R; Weng J; Taveras EM; Redline S Actigraphy-based sleep estimation in adolescents and adults: A comparison with polysomnography using two scoring algorithms. Nat. Sci. Sleep 2018, 10, 13–20. 29403321 10.2147/NSS.S151085 PMC5779275

[52] Tudor-Locke C; Barreira TV; Schuna JM; Mire EF; Katzmarzyk PT Fully automated waist-worn accelerometer algorithm for detecting children’s sleep-period time separate from 24-h physical activity or sedentary behaviors. Appl. Physiol. Nutr. Metab 2014, 39, 53–57. 24383507 10.1139/apnm-2013-0173

[53] Saunders RP; Dowda M; Mciver K; McDonald SM; Pate RR Physical and social contexts of physical activity behaviors of fifth and seventh grade youth. J. Sch. Health 2018, 88, 122–131. 29333648 10.1111/josh.12587 PMC6599639

[54] Bassett DR; John D; Conger SA; Fitzhugh EC; Coe DP Trends in physical activity and sedentary behaviors of United States youth. J. Phys. Act. Health 2015, 12, 1102–1111. 25347913 10.1123/jpah.2014-0050

[55] Kemp BJ; Cliff DP; Chong KH; Parrish AM Longitudinal changes in domains of physical activity during childhood and adolescence: A systematic review. J. Sci. Med. Sport 2019, 22, 695–701. 30630743 10.1016/j.jsams.2018.12.012

[56] Dalene KE; Anderssen SA; Andersen LB; Steene-Johannessen J; Ekelund U; Hansen BH; Kolle E Secular and longitudinal physical activity changes in population-based samples of children and adolescents. Scand. J. Med. Sci. Sports 2018, 28, 161–171. 28299832 10.1111/sms.12876

[57] Harding SK; Page AS; Falconer C; Cooper AR Longitudinal changes in sedentary time and physical activity during adolescence. Int. J. Behav. Nutr. Phys. Act 2015, 12, 44. 25888805 10.1186/s12966-015-0204-6 PMC4391111

[58] Llorente-Cantarero FJ; Aguilar-Gómez FJ; Anguita-Ruiz A; Rupérez AI; Vázquez-Cobela R; Flores-Rojas K; Aguilera CM; Gonzalez-Gil EM; Gil-Campos M; Bueno-Lozano G; Changes in physical activity patterns from childhood to adolescence: Genobox longitudinal study. Int. J. Environ. Res. Public Health 2020, 17, 7227. 33023228 10.3390/ijerph17197227 PMC7579043

[59] Parker K; Timperio A; Salmon J; Villanueva K; Brown H; Esteban-Cornejo I; Cabanas-Sánchez V; Castro-Piñero J; Sánchez-Oliva D; Veiga OL Activity-related typologies and longitudinal change in physical activity and sedentary time in children and adolescents: The UP&DOWN Study. J. Sport Health Sci 2021, 10, 447–453. 33836977 10.1016/j.jshs.2020.02.004 PMC8343008

[60] Dishman RK; McIver KL; Dowda M; Saunders RP; Pate RR Self-efficacy, beliefs, and goals: Moderation of declining physical activity during adolescence. Health Psychol. 2019, 38, 483. 30973746 10.1037/hea0000734 PMC6522322

[61] Miller J; Nelson T; Barr-Anderson DJ; Christop MJ; Winkler M; Neumark-Sztainer D Life events and longitudinal effects on physical activity: Adolescence to adulthood. Med. Sci. Sports Exerc 2019, 51, 663. 30673690 10.1249/MSS.0000000000001839 PMC7166312

[62] Kim Y; Hibbing P; Saint-Maurice PF; Ellingson LD; Hennessy E; Wolff-Hughes DL; Perna FM; Welk GJ Surveillance of youth physical activity and sedentary behavior with wrist accelerometry. Am. J. Prev. Med 2017, 52, 872–879. 28526364 10.1016/j.amepre.2017.01.012 PMC5497761

[63] Hannan K; Hiscock H Sleep problems in children. Aust. Fam. Physician 2015, 44, 880–884. 27054204

[64] Cabré-Riera A; Torrent M; Donaire-Gonzalez D; Vrijheid M; Cardis E; Guxens M Telecommunication devices use, screen time and sleep in adolescents. Environ. Res 2019, 171, 341–347. 30716511 10.1016/j.envres.2018.10.036

[65] Hale L; Guan S Screen time and sleep among school-aged children and adolescents: A systematic literature review. Sleep Med. Rev 2015, 21, 50–58. 25193149 10.1016/j.smrv.2014.07.007 PMC4437561

[66] Lissak G Adverse physiological and psychological effects of screen time on children and adolescents: Literature review and case study. Environ. Res 2018, 164, 149–157. 29499467 10.1016/j.envres.2018.01.015

[67] Tavernier R; Willoughby T Sleep problems: Predictor or outcome of media use among emerging adults at university? J. Sleep Res 2014, 23, 389–396. 24552437 10.1111/jsr.12132

[68] Twenge JM; Hisler GC; Krizan Z Associations between screen time and sleep duration are primarily driven by portable electronic devices: Evidence from a population-based study of US children ages 0–17. Sleep Med. 2019, 56, 211–218. 30639033 10.1016/j.sleep.2018.11.009

[69] Bartel KA; Gradisar M; Williamson P Protective and risk factors for adolescent sleep: A meta-analytic review. Sleep Med. Rev 2015, 21, 72–85. 25444442 10.1016/j.smrv.2014.08.002

[70] Billows M; Gradisar M; Dohnt H; Johnston A; McCappin S; Hudson J Family disorganization, sleep hygiene, and adolescent sleep disturbance. J. Clin. Child. Adolesc. Psychol 2009, 38, 745–752. 20183658 10.1080/15374410903103635

[71] Inhulsen MBM; Busch V; Kalk R; van Stralen MM Adolescent sleep duration: Associations with social-cognitive determinants and the mediating role of sleep hygiene practices. J. Sleep Res 2022, e13774. 36367212 10.1111/jsr.13774

[72] Storfer-Isser A; Lebourgeois MK; Harsh J; Tompsett CJ; Redline S Psychometric properties of the Adolescent Sleep Hygiene Scale. J. Sleep Res 2013, 22, 707–716. 23682620 10.1111/jsr.12059 PMC3752307

